# All-photon logic gate calculation based on phase change materials

**DOI:** 10.1515/nanoph-2025-0155

**Published:** 2025-09-18

**Authors:** Jiapeng Sun, Jinhua Mou, Jiaxing Gao, Jianwei Wang, Yuxin Liu, Wei Jin, Yu Zhang, Lu Liu, Hanyang Li, Zhihai Liu

**Affiliations:** Harbin Engineering University, Harbin 150001, China; Key Laboratory of In-Fiber Integrated Optics, Harbin, China; Ministry of Education, Key Laboratory of Photonic Materials and Device Physics for Oceanic Applications, Ministry of Industry and Information Technology of China, Harbin Engineering University, Harbin, China

**Keywords:** logic gate computation, multi-level memory, non-volatile, phase change material

## Abstract

Information modulation plays a crucial role in the contemporary development of photonic information networks. However, prevailing optical modulators exhibit challenges such as structural complexity, high cost, elevated insertion losses, and crosstalk issues in optoelectronic conversion. To address these concerns, we propose a novel all-photonic non-volatile fiber device based on Ge_2_Sb_2_Te_5_ (GST), integrating a dual peanut-shaped microstructure and GST. The state of GST is manipulated through external laser modulation, enabling repeatable or randomly accessible ten-level data storage. The all-photonic modulator features non-volatility, a contrast ratio of 21 dB, and repeatability (0.2 dB). Furthermore, by configuring the all-photonic modulator in both series and parallel connections, we successfully implement logical gate functions “AND” and “OR”, showcasing significant potential in information network control.

## 1 Introduction

Over the past two decades, the development and technological advancements of an all-photonic information network have gained increasing attention. The optimization of an all-photonic network primarily focuses on improving both the information transmission rate and the enhancement of information modulation [1], [2], [3]. Currently, the developed high-speed information transmission optical fibers can transmit data at 99.7 % of the speed of light, resulting in a data rate of 73.7 Tb/s [4]. This speed is over a thousand times faster than commonly used 40 Gb-level optical fiber cables, significantly reducing transmission latency [5], [6]. Therefore, the optimization of information modulation has become a top priority in the construction of an all-photonic information network.

It is noteworthy that significant achievements have been made in the field of information modulation for all-photonic information networks with devices such as AlGaAs microwave modulators [7], silicon nitride micro-ring modulators [8], [9], VO_2_ spherical phase modulators [10], lithium niobate acousto-optic modulators [11], [12], photonic logic computing unit [13], [14], [15] and photonic crystal optical modulators [16], [17], [18]. However, these optical modulators still exhibit significant drawbacks [19]. These optical modulators face challenges such as the need for a constant energy supply to maintain modulation states, sensitivity to temperature variations, low repetition rates, and susceptibility to element separation, which continue to impede the development of the all-photonic information network [8], [20], [21], [22].

The emergence of phase-change materials (PCMs) has provided an opportunity to overcome these limitations. In recent years, phase-change materials have garnered attention due to their high contrast in electrical and optical properties between crystalline and amorphous phases, long-term retention in various intermediate states without additional energy maintenance, and high endurance with up to 10^15^ switching cycles [23], [24], [25], [26], [27]. Particularly, Ge_2_Sb_2_Te_5_ (GST) stands out as a PCM with outstanding performance in terms of switching speed and stability [28], [29], [30], [31]. By adjusting the power and pulse count of the modulating light source, on-chip multi-level non-volatile storage with a response speed below 100 ns can be achieved [32], [33]. Furthermore, the variation in the refractive index of GST can be exploited to achieve an all-photonic phase modulation based on silicon-based waveguides [34], [35], [36], [37]. Additionally, combining GST with different photonic structures enables the assignment of “weights” to synaptic connections in neural networks, allowing for the determination of event priorities [38], [39], [40], [41], [42]. Although these optical modulators have advantages such as small size, complex functionality, and high integration, all-optical modulators based on lithography face challenges such as high cost, complex processes, and low ubiquity, making them difficult to be compatible with the current all-photonic information network [43], [44]. With the recent development of information networks in recent years, it is evident that optical fibers are an excellent information carrier [45], [46], [47], [48]. Therefore, we attempt to combine optical fiber processing with GST to realize the construction of a novel all-photonic fiber modulator [49], [50], [51], [52].

**Figure 1:**
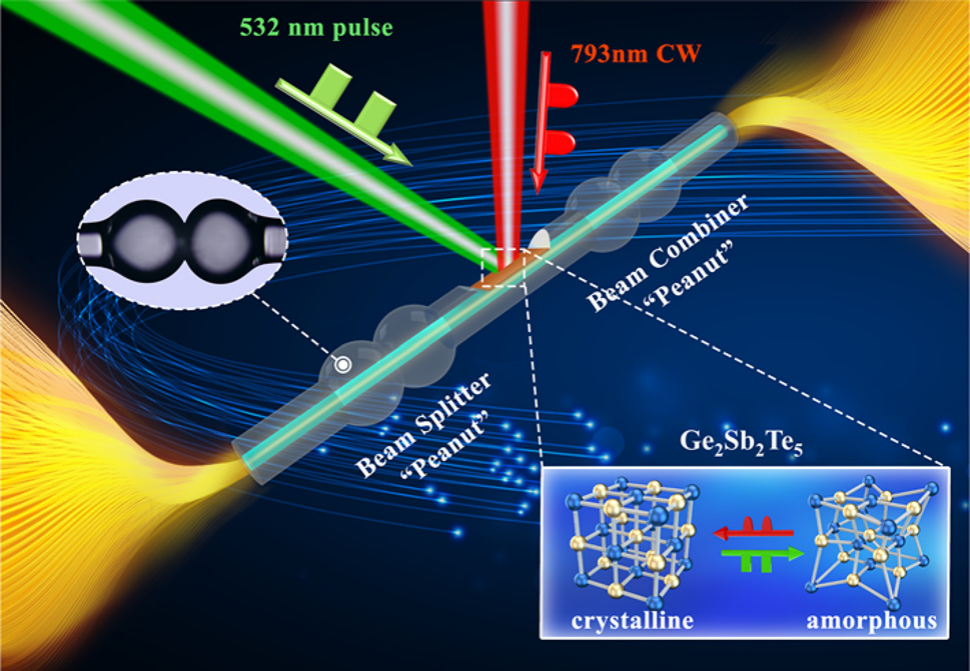
Working principle of the all-optical modulator. The modulator employs a dual peanut-shaped optical fiber structure and GST composition. During the modulation process, modulation information is stored in the GST film coated on the D-shaped side-polished region. The multi-level modulation of the all-optical modulator is accomplished by irradiating the GST film with a 532 nm pulsed laser and a 793 nm CW laser.

In this paper, we propose and demonstrate an all-optical modulator based on a dual-peanut-shaped optical fiber microstructure and GST (Figure 1). It is noteworthy that to enhance the modulation effect of GST on the internal optical signals within the optical fiber microstructure, we utilize a carbon dioxide laser for D-shaped side-etching between the two peanut structures and apply GST to the side-etched area using magnetron sputtering. To achieve ten-level information modulation, a 532 nm pulsed laser and a 793 nm continuous (CW) laser are employed for information storage and erasure, respectively. Besides, our information modulator features a 21 dB extinction ratio, high repeatability (0.2 dB), and the capability to randomly access any modulation level. Furthermore, by connecting peanut-shaped all-optical modulators in series and in parallel, we achieve the logical gate functions “AND” and “OR”. This dual-peanut-shaped all-optical modulator overcomes the von Neumann bottleneck and the crosstalk in optoelectronic conversion, showcasing significant potential in information network control.

## 2 Construction and characterization of optical switch

Our all-optical modulator is fabricated from single-mode optical fiber (SMF-28). A fiber fusion splicer (Notevio NT-200s) is used to discharge the flat end face of the optical fiber. Under the action of high-voltage electric arcs, the end face of the optical fiber melts, and due to the presence of surface tension, it gradually forms a smooth fiber ball. Subsequently, a low-voltage discharge arc is used to connect two fiber balls, forming a peanut-shaped coupler. Connecting two peanuts in series creates a Mach–Zehnder interferometer (MZI).

**Figure 2:**
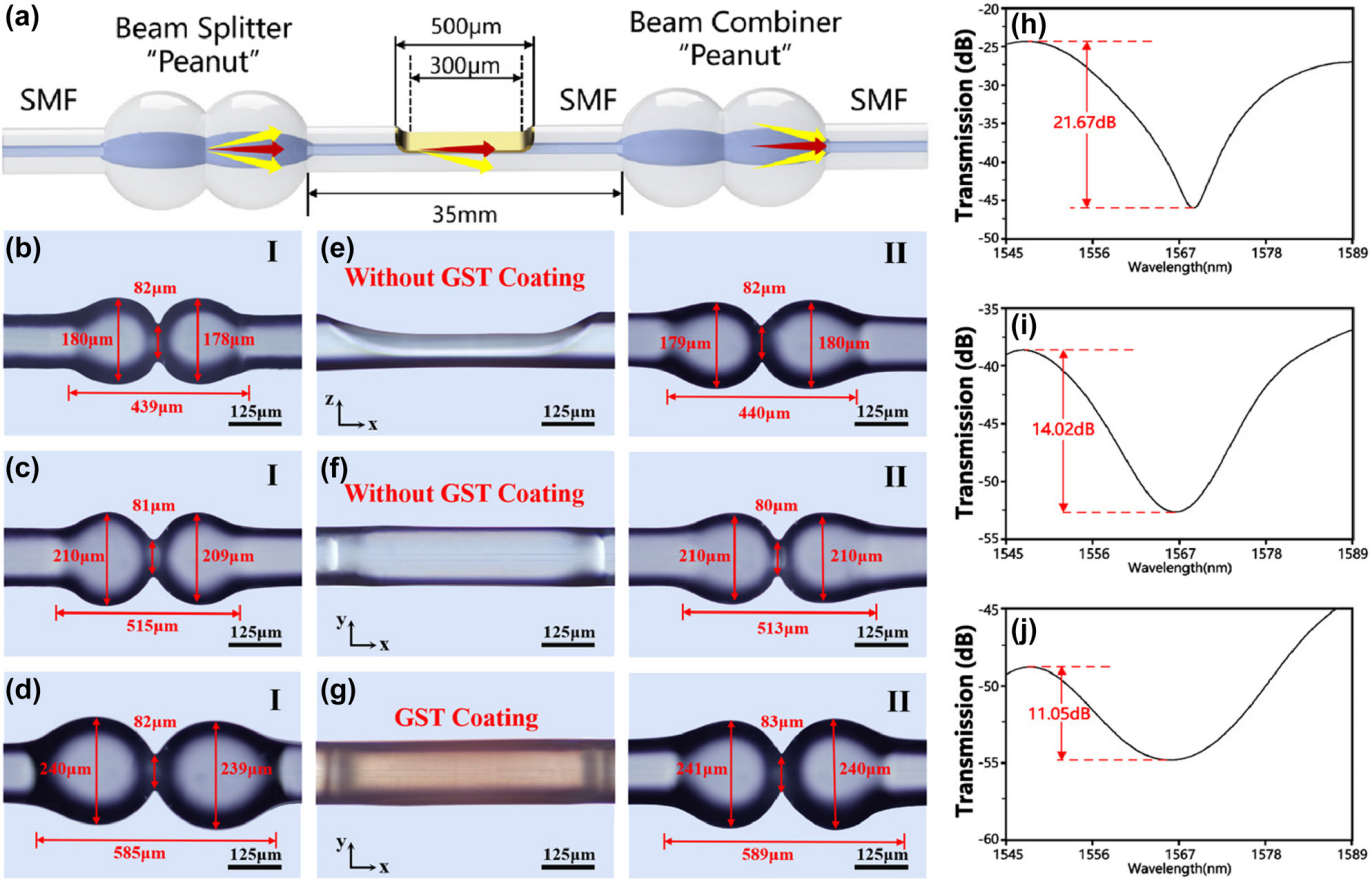
Performance and characterization of dual peanut-shaped structure. (a) Schematic diagram illustrating the mechanism of the dual peanut-shaped Mach–Zehnder interferometer (MZI). (b–d) Microscopic images of peanut-shaped MZIs with diameters of 180 μm, 210 μm, and 240 μm, where the lengths of the “peanuts” are 440 μm, 515 μm, and 585 μm, respectively. Microscopic images of the D-shaped side-polished region without GST coating in the *x*–*z* plane (e) and *x*–*y* plane (f). (g) Microscopic images of the D-shaped side-polished region with GST coating. (h–j) Transmission spectra of the three peanut-shaped MZIs (*d* = 180 μm, 210 μm, and 240 μm), with interference intensities of 21.67 dB, 14.02 dB, and 11.05 dB, respectively.

In the MZI, light travels along the core of the optical fiber to reach the first “peanut beam splitter,” where light in the core is coupled into the cladding. The optical signal in the core continues to propagate, while the light in the cladding is modulated by GST when passing through the side-etching region. As the light in the cladding and the core passes through the second “peanut coupler,” the light in the cladding is coupled back into the core, causing interference with the light in the core, as shown in Figure 2(a). It is noteworthy that, to increase the interaction area between the optical signal inside the optical fiber and GST, we use a high-frequency carbon dioxide laser to side-etching the interference arm region between the two peanuts. Additionally, a 45 nm-thick layer of GST film is coated in the D-shaped side-etching region using magnetron sputtering. Figure 2(e)–(g) illustrate microscope images of the D-shaped side-etching region in the states of “no GST coating” along the *x*–*z* direction, *x*–*y* direction, and with GST coating (*x*–*y* direction).

In this peanut MZI, *φ*_cl_ and *φ*_core_ are phases of the cladding mode and core mode:

(1) $$\varphi_{\text{cl}}=\frac{2\pi \times n_{\text{eff}}^{\text{cl}}\times L}{\lambda_{0}}$$

(2) $$\varphi_{\text{core}}=\frac{2\pi \times n_{\text{eff}}^{\text{core}}\times L}{\lambda_{0}}$$

where *λ*_0_ is the central wavelength, $n_{\text{eff}}^{\text{cl}}$ is the effective refractive index of the cladding structure, and $n_{\text{eff}}^{\text{core}}$ is the effective refractive index of the core structure, and *L* represents the interaction length of the interference arm between the two peanut structures. In our peanut MZI, the optical path difference between the two models can be approximated by:

(3) $$\Delta_{p}=\underset{L}{\int }\left(n_{\text{eff}}^{\text{cl}}-n_{\text{eff}}^{\text{core}}\right)d_{z}$$

Here, the transmission intensity of our peanut MZI can be defined as:

(4) $$I=I_{1}+I_{2}+2\sqrt{I_{1}+I_{2}}\cos\left(\frac{2\pi L\times \left(n_{\text{core}}-n_{\text{cl}}\right)}{\lambda_{0}}\right)$$

(5) $$I=I_{1}+I_{2}+2\sqrt{I_{1}+I_{2}}\cos\left(\frac{2\pi \Delta_{p}}{\lambda_{0}}\right)$$

where *I*, *I*_1_, and *I*_2_ are the transmission intensities of the light in the optical path, cladding, and core, respectively. In our MZI measuring system, the transmission intensity and phase shift can be monitored by a high-accuracy optical spectrum analyzer (OSA, AQ6317C, Yokogawa TM resolution of 0.2 nm).

To investigate the impact of peanut diameter on the performance of the all-optical modulator, devices with diameters of 180 μm, 210 μm, and 240 μm were fabricated, as shown in Figure 2(b)–(d). The lengths of the peanut structures (along the *x*-axis) are 440 μm, 515 μm, and 585 μm, respectively. An ASE broadband light source is used as the probing light, and an OSA is employed to detect the spectra of peanut-shaped interferometers with different diameters, as shown in Figure 2(h)–(j). From the graphs, it can be observed that with the increase in peanut diameter, the interference intensity of the peanut-shaped MZI decreases from 21.67 dB to 11.05 dB. In this study, to obtain a distinctly discernible intermediate state, the interference intensity of the peanut-shaped MZI should be as large as possible. Therefore, we choose the peanut-shaped MZI with a diameter of 180 μm as the core structure for the all-optical modulator.

## 3 Theoretical and simulation analysis

**Figure 3:**
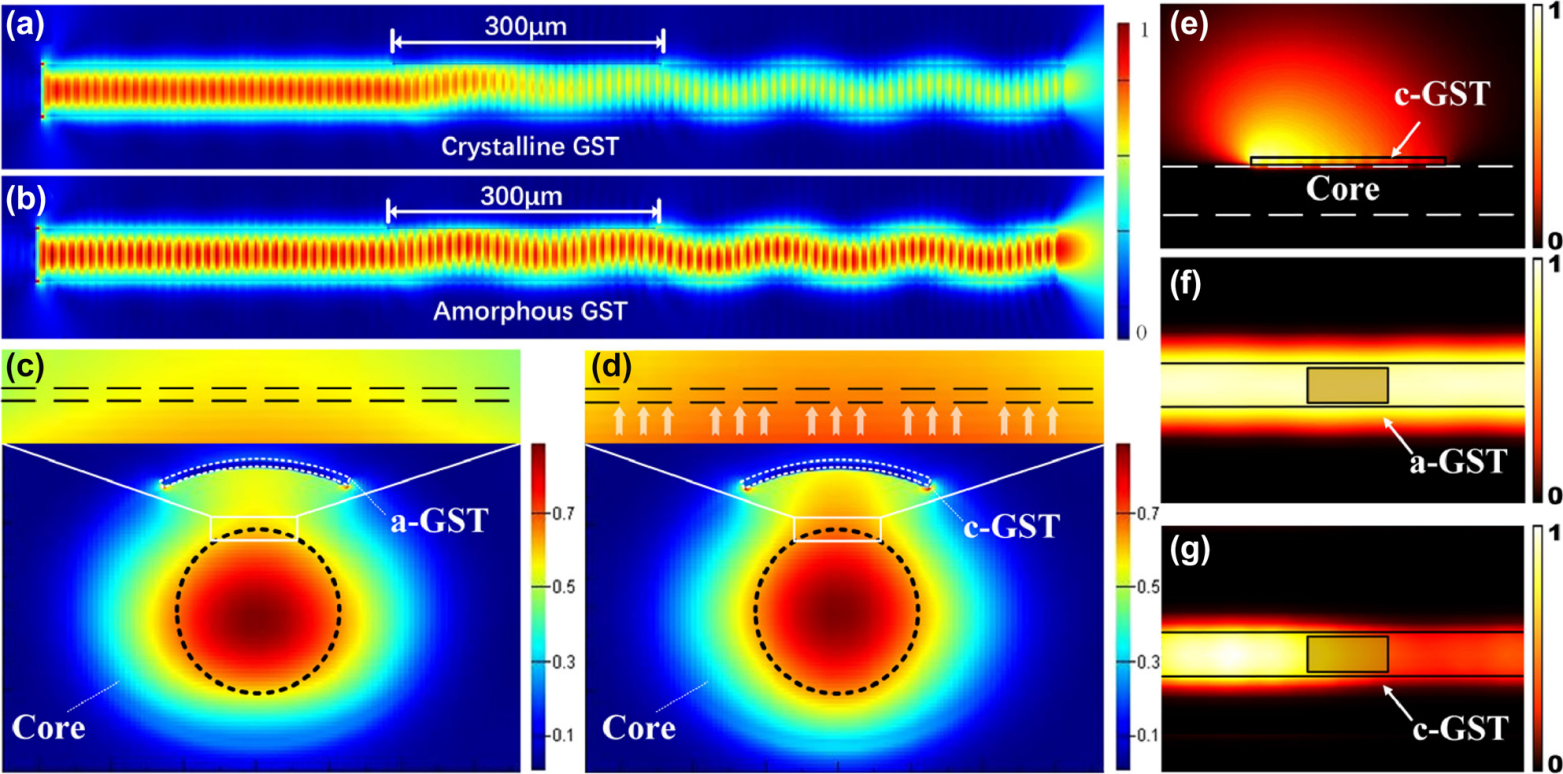
Simulation of phase-change storage unit. (a–b) Distribution of electric fields in the crystalline, amorphous, and intermediate states of GST. The results indicate that GST can block optical signals within the optical fiber, showing distinct behavior in its two states. (c–d) Simulation of the electric field interface distribution in the D-shaped side-polished region coated with GST material. When GST is in the crystalline state, the energy distribution of the electric field shifts towards c-GST. This suggests that, compared to a-GST, c-GST exhibits a stronger attraction to optical signals. (e) Thermal field distribution in the crystalline state of the D-shaped side-polished region with GST, showing thermal attenuation along the direction of optical signal transmission. (f–g) Thermal field distribution in the D-shaped side-polished region with GST in amorphous and crystalline states, respectively.

To investigate the influence of GST in different states on the all-optical modulator, we measured the negative refractive index of both crystalline and amorphous GST using an ellipsometer. Additionally, we employed the time-domain finite element method to simulate the fiber side-etching region. The refractive index of GST was set to the crystalline state (*n*__c-GST_ = 6.5 + 1.1*k*), and the simulated light field distribution is shown in Figure 3(a). As illustrated in Figure 3(b), when the negative refractive index of GST changes to the amorphous state parameters (*n*__a-GST_ = 3.9 + 0.02*k*), a significantly different change occurs in the optical signal inside the fiber. It can be observed that when GST is in the crystalline state, the extinction coefficient of GST increases, and there is significant attenuation of light in the side-etching region. Furthermore, simulations of the light field in the cross-section of the optical side-etching region were conducted. Comparing Figure 3(c) and (d), when GST is in the amorphous state, the light field in the fiber cross-section is concentrated in the central region because the extinction coefficient of GST in the amorphous phase is significantly smaller, and the core mode is undisturbed. As GST transitions to the amorphous state, its extinction coefficient increases, the optical signal inside the fiber is absorbed by GST, and simultaneously, the light field undergoes a shift toward the GST-coated plane.

Subsequently, we established a three-dimensional model to investigate the temperature characteristics of GST in the operational scenario of the all-optical modulator. The initially crystalline GST coated on the side-etching region was set. As seen in Figure 3(e), the optical signal, upon contact with GST, is absorbed, generating a significant amount of heat that attenuates along the direction of GST. It is observed that the strong absorption effect of crystalline GST leads to the generation of a large amount of heat in the part of GST that first comes into contact with light. This results in the occurrence of “element separation,” “worm surface,” and “surface GST oxidation.” All of which significantly reduce the lifespan of the all-optical modulator. Therefore, we coated a uniform layer of ITO film on the surface of the GST film. The ITO film serves as both a uniform heat-conducting layer and an anti-oxidation layer. Figure 3(f) and (g) show the internal heat distribution of the fiber when GST is in the amorphous and crystalline states, respectively. In particular, the heat distribution trend in the GST-coated region in Figure 3(g) is consistent with that in Figure 3(e).

## 4 Experimental

**Figure 4:**
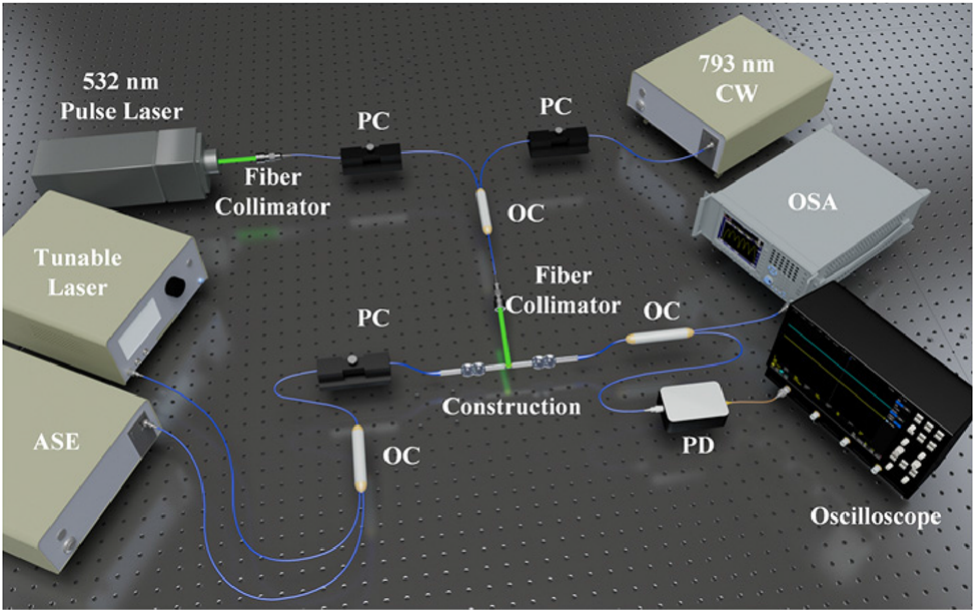
Optical modulation system setup for the all-optical modulator. An amplified spontaneous emission (ASE) source (A-0002, wavelength: 1,152.5–1,570 nm; Hoyatek Co., Ltd.) is connected to one end of the dual peanut-shaped MZI via a polarization controller (PC). The other end of the dual peanut-shaped MZI is connected to an optical spectrum analyzer (OSA, Yokogawa AQ6370C) with a resolution of 0.02 nm to record the modulated optical signal. A tunable laser source (TLS, Agilent 81940A) and a photodetector (PD, New Focus, Model 2011) are connected to a high-speed digital oscilloscope (DSO, Dggint MSOX-2022A) for monitoring the phase changes in the dual peanut-shaped MZI. The 532 nm pulsed laser and 793 nm CW laser used for modulating GST are coupled into a fiber collimator via a 2 × 1 coupler after passing through the PC, and they irradiate the GST film to achieve modulation.

**Figure 5:**
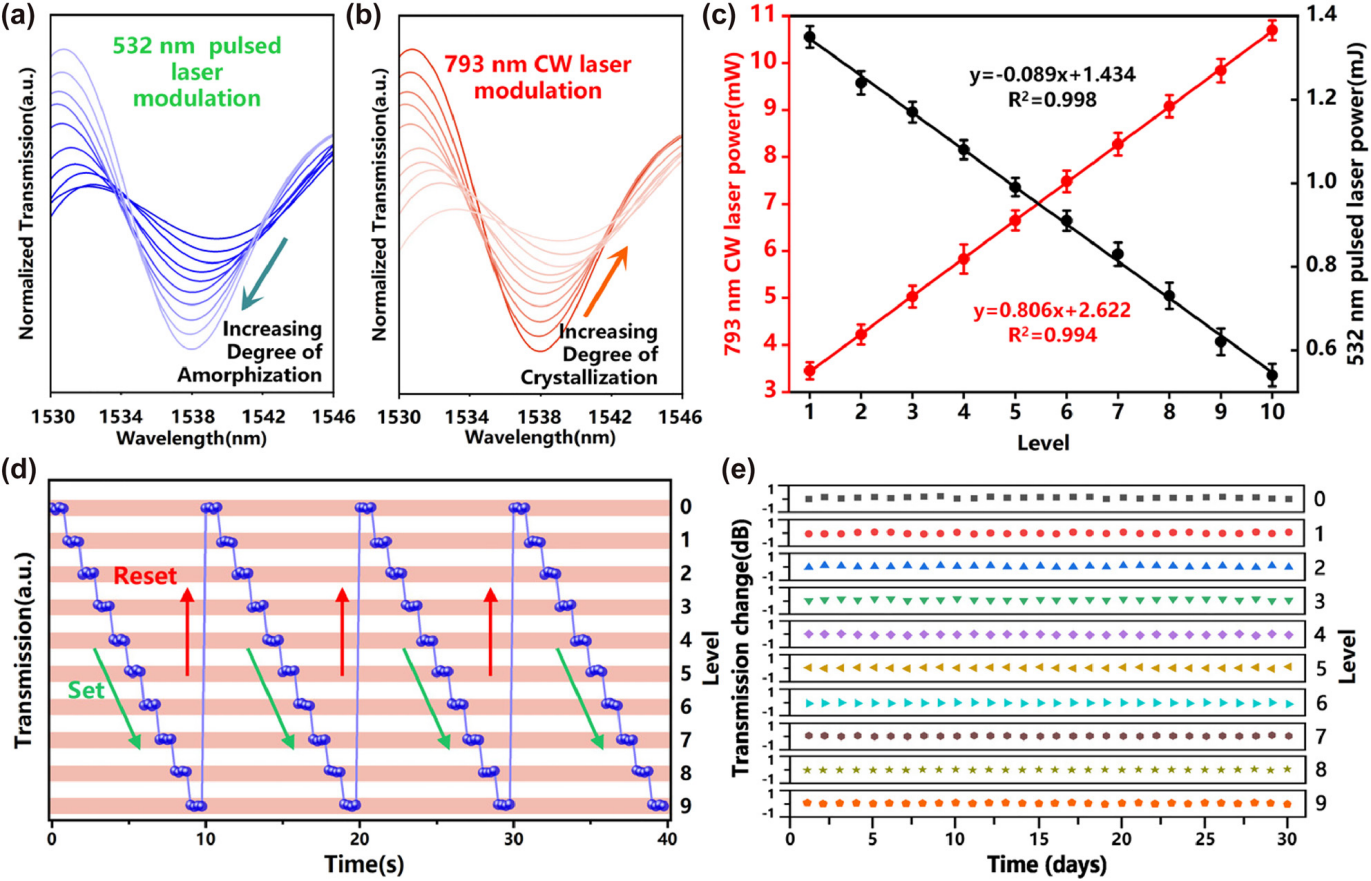
Multilevel modulation of phase-change storage unit. (a–b) Ten-level transmission spectra of GST with varying degrees of amorphization (crystallization) after irradiation with a 532 nm pulsed laser and a 793 nm CW laser, respectively. (c) Relationship between the output power of the 793 nm CW laser (red line) and the energy of the 532 nm pulsed laser with respect to the modulation level. (d) Independent multi-level states are achieved through probe modulation during four repetitive switching cycles. (e) Intensity fluctuations at different modulation levels over 30 days.

To achieve multi-level modulation functionality of the all-optical modulator, a 532 nm pulsed laser and a 793 nm CW laser were used to alternately irradiate the GST film coated on the side-etching region in a D-shaped configuration, as shown in Figure 4. Signal light (HY-ASE-C + L-N-17-BD-FA, CONNECT) and a frequency-swept laser source are sent from the two ports of the first 2 × 1 coupler to a polarization controller (PC), undergoing interference and modulation through the dual-peanut-shaped MZI. An optical spectrum analyzer (OSA) and oscilloscope are employed to monitor the optical signal at the output end of the dual-peanut-shaped MZI. In Figure 5(a) and (b), it can be observed that during the modulation process of the initially crystalline GST film using a 532 nm pulsed laser, as the degree of amorphization of GST increases, the refractive index and extinction coefficient of GST decrease. This leads to a reduction in the effective refractive index in the cladding region and, simultaneously, a decrease in GST’s absorption of light. As a result, the interference peak transmittance of the peanut-shaped MZI increased by 3.3 dB, the extinction ratio increased from 1.3 dB to 7.1 dB, and the transmission spectrum exhibited a blue shift of 1.44 nm.

Conversely, when modulating with a 793 nm CW laser, the degree of crystallization of GST increases, leading to an increase in the extinction coefficient and refractive index of GST. This results in an increase in the effective refractive index in the fiber cladding region, intensifying light absorption by GST. Consequently, the transmission spectrum projection intensity of the MZI decreased by 3.7 dB, the extinction ratio decreased from 6.9 dB to 0.8 dB, and the transmission spectrum underwent a redshift of 1.18 nm. Thus, we have achieved the reversible transformation from the “ON” state of “1” to the “OFF” state of “0” in the all-optical modulator.

In Figure 5(c), we present the relationship between the modulation level in the ten-level modulation and the output power of the 793 nm CW laser and the 532 nm pulsed laser, respectively. We observed a correspondence between the output energy of the modulating laser and the modulation levels of GST. When using a 532 nm pulsed laser to induce a ten-level transition of GST from the crystalline state to the amorphous state, the laser output energy levels were 1.35, 1.24, 1.17, 1.08, 0.99, 0.91, 0.83, 0.73, 0.62, and 0.54 mJ, respectively. Similarly, to achieve a ten-level transition from the “ON” state to the “OFF” state in the all-optical modulator, the output power of the 793 nm CW laser was set to ten different levels: 3.45 ± 0.18, 4.22 ± 0.21, 5.03 ± 0.23, 5.83 ± 0.31, 6.65 ± 0.21, 7.48 ± 0.24, 9.08 ± 0.23, 9.84 ± 0.25, 10.70 ± 0.21 mW. This relationship between modulation levels and laser output power facilitates the sequential, reverse, and random modulation of the all-optical modulator. To verify the repeatability of the ten-level all-optical modulator, we conducted four consecutive, complete cycles, each including the crystalline state, amorphous state, and eight stable and distinctly distinguishable intermediate states. As shown in Figure 5(d), under the condition of no external energy maintenance, the all-optical modulator can repeatedly switch between different energy levels, with the transmission intensity differences between the same levels much smaller than those between different levels. Additionally, I conducted stability tests on the all-optical modulator. Over a continuous period of 30 days, we measured and recorded the transmission intensity of 10 energy levels each day, as shown in Figure 5(e). The fluctuation in transmission intensity for each energy level of the all-optical modulator was less than 0.2 dB over the 30-day period. Therefore, the experimental results demonstrated that our all-optical modulator has a very significant non-volatility.

**Figure 6:**
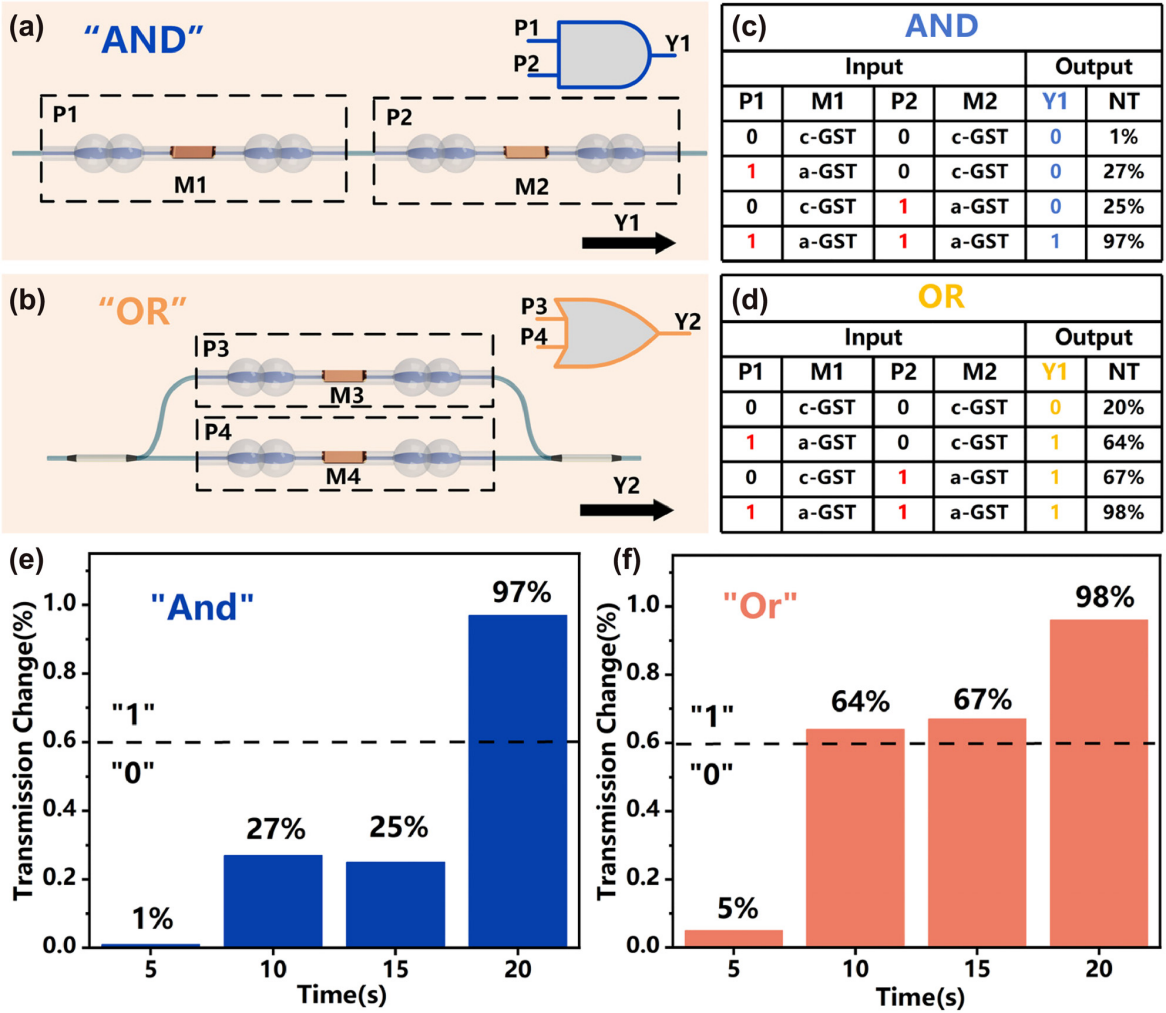
AND/OR logic computation. (a–b) Schematic representation of the mechanisms of the AND and OR logic gates. The logical AND (OR) operation is achieved by serially connecting (paralleling) two all-optical modulators. (c–d) Truth tables for basic logic AND (OR) operations. Outputs for four different input state combinations (00, 01, 10, 11): “0, 0, 0, 1” (0, 1, 1, 1). (e–f) Normalized transmittance output plots for different input combinations of the modulo-2 logic AND (OR) operations. The logic calculation output NT is above (below) the reference value for logic “1” (“0”).

Additionally, we attempted to use the all-optical modulator in the aspect of logic gate computation. Initially, two all-optical modulators, P1 and P2, were connected in series to achieve the “AND” operation, as shown in Figure 6(a). M1 and M2 were defined as the states of GST coated in the side-casting region. It is worth noting that the “AND” gate developed in this work is based on binary operations, so in this study, GST exists in only two states: crystalline (c-GST) and amorphous (a-GST). The transmittance output value of this “AND” gate can be represented as *T*_a_ = P1 × P2. To reduce computational errors, GST was modulated to the amorphous state before logic calculations. We used a 532 nm pulse laser with a pulse width of 10 nm and a power of 1.35 mJ, and a 793 nm CW laser with an energy of 3.45 mW to assign the digital “1” and “0”, respectively. We used an ASE light source and a swept laser to read information from the logic gate. We set the reference line to a normalized transmittance (NT) of 60 % (ref = 60 %). When the input terminals of the “AND” gate were given “00”, “10”, “01”, and “11”, the NT at the output terminals of the “AND” gate were “1 %”, “27 %”, “25 %”, and “97 %”, respectively. Therefore, the logical operation results for the four inputs were “0”, “0”, “0”, and “1”, as shown in Figure 6(c) and (e). Similarly, we connected two all-optical modulators, P3 and P4, in series to achieve the “OR” operation, as shown in Figure 6(b). We used two lasers to modulate M3 and M4 to change the input values of the “OR” gate. The transmittance output value of this “OR” gate can be represented as *T*_o_ = P3 + P4. As shown in Figure 6(d) and (f), when the input terminals of the “OR” gate were given “00”, “10”, “01”, and “11”, the NT at the output terminals of the “OR” gate were “5 %”, “64 %”, “67 %”, and “98 %”, respectively. Comparing the output NT values with the reference line, the logical operation results for the four inputs were “0”, “1”, “1”, and “1”.

## 5 Conclusions

In conclusion, we have presented, for the first time, a fully optical modulator with ten-level controllability, combining GST and a peanut-shaped MZI fiber microstructure. The interference intensity of the MZI can reach 21 dB. External laser modulation using a 532 nm pulse laser and a 793 nm CW laser was employed to adjust the crystalline state of GST. Four consecutive complete cycles were used to demonstrate the significant repeatability of the all-optical modulator. Through continuous monitoring of each level for 30 days at a daily frequency, it was observed that the transmission intensity fluctuations of the all-optical modulator were less than 0.2 dB over the 30-day period, indicating excellent non-volatility. Furthermore, the serial and parallel connection of two all-optical modulators achieved “AND” and “OR” computations. Undoubtedly, this study demonstrates the remarkable capability of a multi-level all-optical modulator with long-distance transmission capacity. This fiber-based all-optical modulator can perform more complex calculations, laying the foundation for high-speed data communication and information exchange in future all-optical networks.
